# Influence on Implant Bone Healing of a Collagen Membrane Placed Subjacent the Sinus Mucosa—A Randomized Clinical Trial on Sinus Floor Elevation

**DOI:** 10.3390/dj10060105

**Published:** 2022-06-08

**Authors:** Akihiro Morimoto, Nobuhiro Kobayashi, Mauro Ferri, Giovanna Iezzi, Adriano Piattelli, Natalia Fortich Mesa, Daniele Botticelli

**Affiliations:** 1 Department of Oral Implantology, School of Dentistry, Osaka Dental University, Osaka 573-1144, Japan; stylish.for.life0420@gmail.com (A.M.); n_kobayashi_3009@yahoo.co.jp (N.K.); 2 ARDEC Foundation, Cartagena de Indias 130001, Colombia; medicina2000ctg@hotmail.com; 3 Department of Medical Oral and Biotechnological Sciences, University of Chieti-Pescara, 66100 Chieti, Italy; gio.iezzi@unich.it (G.I.); apiattelli@unich.it (A.P.); 4 School of Dentistry, University Corporation Rafael Núñez, Cartagena de Indias 130001, Colombia; natalia.fortich@curnvirtual.edu.co; 5 ARDEC Academy, 47923 Rimini, Italy

**Keywords:** bone grafting, xenograft, dental implants, bone healing, osteoconductivity, sinus mucosa, morphometry, collagen membrane

## Abstract

Background: Perforation of the sinus mucosa is quite a frequent complication that might occur during sinus floor elevation. The perforation is often protected with a collagen membrane to avoid the extrusion of graft particles within the sinus. However, this procedure might hinder the innate osteogenic potential of the sinus mucosa. Hence, the aim of the study was to evaluate the influence of a placement of a collagen membrane subjacent the Schneiderian membrane during sinus floor elevation on implant bone healing. Methods: Twenty volunteers took part in the trial. Ten were randomly included in the group that received a collagen membrane subjacent the sinus mucosa (Mb group), and ten did not receive the membrane (non-Mb group). A collagenated corticocancellous porcine bone was used to fill the elevated space. Six 6 months after the sinus floor elevation, a mini implant was placed transcrestally and retrieved after a further 3 months. Histological analyses were then performed on the full body of the mini implant as well as on its coronal and apical portions. Results: The new bone apposition proportion onto the implant surface was similar in the Mb and non-Mb groups, both in the apical and coronal portions of the mini implants. A lesser amount of graft was found in contact with the surface. New bone density around the mini implants were similar both in the apical and coronal portions. However, a statistically higher proportion of graft particles was found in the Mb group compared to the non-membrane group. Conclusions: The placement of a collagen membrane subjacent the sinus mucosa did not affect bone healing at implants and bone density.

## 1. Introduction

Sinus floor elevation is a well-documented procedure aiming to increase the bone volume in the posterior regions of the maxilla. It has been shown that this procedure allows a high success rate over time [1,2]. The perforation of the sinus mucosa is quite a common complication that might occur during the surgical procedures with a frequency ranging between 5% and 55% [3,4,5,6,7]. Small perforations might be left without treatment [8], while larger perforations might be sutured [4,9,10], closed with fibrin glue [4,9,11], or protected with a collagen membrane [6,7,12]. The sinus membrane has been shown to have the potential of producing bone [13,14]. According to this observation, the placement of a collagen membrane subjacent the sinus mucosa might reduce its own osteogenic potential. The effect of the placement of a collagen membrane subjacent the sinus mucosa on bone formation within the elevated space was evaluated in animal studies, and no differences were seen between the groups with and without membranes [15,16,17]. Moreover, no new bone formation from the sinus mucosa was observed during the early healing after sinus floor elevation in monkeys [18]. The influence of a membrane placed subjacent the sinus mucosa on dimensional changes of the augmented maxillary sinus floor was also evaluated on CBCTs [3]. No differences were found after nine months of healing compared to the control sites without membranes. The effect on bone healing at implants installed in augmented sinuses after healing has not been evaluated yet. Hence, the aim of the present study was to evaluate the influence of a collagen membrane placed subjacent the Schneiderian membrane during sinus floor elevation on bone healing around implants. The null hypothesis was that the placement of a collagen membrane subjacent the sinus mucosa will not affect bone healing and implant integration.

## 2. Materials and Methods

### 2.1. Ethical Statement

The protocol was approved by the Ethical Committee of the University Corporation Rafael Núñez, Cartagena de Indias, Colombia (protocol #03-2015; 4 December 2015). All clinical procedures were performed in the same university. The Declaration of Helsinki on medical protocols and ethics was strictly followed. All participants were thoroughly informed about procedures and possible complications, and they signed informed consent to participate in the study. The article was written following the indications of the CONSORT checklist.

The RCT was registered at ClinicalTrials.gov with the following identifier code: NCT03902457.

### 2.2. Study Population

The following inclusion criteria were adopted: (i) need for a fixed prosthetic rehabilitation at edentulous zones in the posterior maxilla; (ii) height of the sinus floor ≤4 mm; (iii) ≥21 years of age; and (iv) not pregnant. The following exclusion criteria were adopted: (i) contraindications for oral surgical procedures; (ii) undergoing chemotherapic or radiotherapeutic treatment; (iii) presence of acute or chronic sinusitis; (iv) previous bone augmentation procedures in the region; (v) >10 cigarettes per day; and (vi) patients on bisphosphonates.

### 2.3. Study Design

Sinus floor elevation was performed, and before the placement of the biomaterial, a collagen membrane with standardized dimensions (9 × 13 mm) was placed only at the test sites subjacent the sinus mucosa, in a central position in respect to the osteotomy. No membranes were placed in the control sites. The access osteotomies were covered by collagen membranes in both groups. Mini implants were placed in the alveolar crest in the elevated region after 6 months from the first surgery and retrieved after a further 3 months. CBCTs were taken before sinus floor elevation and after 1 week and 9 months. The results from the tomographic evaluations are reported elsewhere [3], while the histological evaluations are reported in the present study.

### 2.4. Devices and Biomaterials

A type IV titanium screw-shaped mini implant (Sweden & Martina, Due Carrare, Padua, Italy), 2.4 mm in diameter and 8 mm long, was installed six months after sinus floor elevation and retrieved 3 months after. The surface of the mini implants was sandblasted at 5 atms with zirconia microspheres (grit dimension 120 µm) and subsequently acid etched with hydrofluoric acid followed by immersion in a solution of sulfuric acid and hydrochloric acid (ZirTi surface, Sweden & Martina, Due Carrare, Padua, Italy).

A collagenated corticocancellous porcine bone granule (Gen-Os, 250–1000 μm, OsteoBiol, Tecnoss, Giaveno, Italy) was used to fill the subantral space.

Equine collagen membrane (Evolution, 0.3 mm, OsteoBiol, Tecnoss, Giaveno, Italy) was placed both subjacent the sinus mucosa at the test sites and to cover the antrostomy at both test and control sites.

### 2.5. Sample Size

The sample size for the present RCT study was based on the tomographic evaluations as it was reported in a previous article [3]. A further evaluation that included biopsies of mini implants was also included in the study. An analysis was performed of the histological data from previous RCTs in which the biomaterial and mini implants used were similar to the present study [19,20]. However, it failed to suggest a reasonable sample of patients to be included. In the absence of previous data that compared bone healing at implants installed into augmented sinuses in which a collagen membrane was placed subjacent the sinus mucosa, a difference in bone-to-implant contact percentage (BIC%) of 10% was used for statistical evaluation. A sample of 10 subjects for each group was calculated to be sufficient to disclose differences between the two surfaces in bone-to-implant contact with a ratio of 1 for the control-to-experimental patients, a power of 0.8, an α error of 0.05, and a standard deviation of 7% (PS Power and Sample Size Calculations, Version 3.1.2, by William D. Dupont and W. Dale Plummer, Jr. licensed under a Creative Commons Attribution-NonCommercial-NoDerivs 3.0 United States License).

### 2.6. Randomization and Allocation Concealment

The randomization for the allocation treatment (membrane placed subjacent the sinus mucosa) was performed electronically by an author not involved in the sinus floor elevation and a mini implant installation (M.F.). The treatment assignments were kept in sealed opaque envelopes that were opened at the time of sinus floor elevation.

### 2.7. Clinical Procedures

Detailed descriptions of the surgical procedures were reported in previous articles (Hirota; Imai) [19,20]. In brief, an osteotomy was prepared on the lateral wall of the maxillary sinus using a sonic-air surgical instrument (Sonosurgery^®^ TKD, Calenzano, FI—Italy). The sinus mucosa was elevated, and a collagen membrane with standardized size (9 × 13 mm) was placed subjacent the sinus mucosa at the test sites, while no membranes were used at the control sites. A collagenated corticocancellous porcine bone graft was used to fill the subantral space, and both osteotomies were covered by collagen membranes. After 6 months of healing, a mini implant was placed in the elevated space. Biopsies containing the mini implants were retrieved after a further three months using a trephine (GA33M, Bontempi Strumenti Chirurgici, San Giovanni in Marignano, RN, Italy), 3.5 mm and 4 mm of internal and external diameters, respectively. The trephine was used in an eccentric mode aiming to reduce the dimension of the osteotomy and, at the same time, to obtain sufficient bone volume for histological analysis [21]. The small dimensions of the trephine allowed the immediate insertion of a standard implant (Premium, Sweden & Martina, Due Carrare, Padua, Italy) in the same position.

### 2.8. Histological Preparation of the Biopsies

Aiming to avoid damages, the biopsies were not removed from the trephines. Following the fixation in 10% buffered formalin, the biopsies were dehydrated in an ascending series of alcohol, included in resin (Technovit^®^ 7200 VLC; Kulzer, Wehrheim, Germany) and polymerized. Histological slides, ~30 µm in width, were subsequently prepared following the longitudinal axis of the mini implant. Acid fuchsine and toluidine blue were used as staining.

### 2.9. Histomorphometric Evaluation

The histological slides were coded so that the treatment locations could not be disclosed during the histomorphometric evaluations, which were performed by an expert assessor (D.B.). The software NIS-Elements D 5.11.01 (Laboratory Imaging, Nikon Corporation, Tokyo, Japan) was used for histomorphometric measurements that were performed in an Eclipse Ci microscope (Nikon Corporation, Tokyo, Japan). Measurements were performed in the most coronal and most apical 4 mm of the implant. Data from the full surface and for the two regions were reported separately.

All measurements were conducted at ×200 magnification from the most coronal and most apical contact of the bone to the implant surface. New bone, pre-existing (old) bone, residual graft, and soft tissues (bone marrow and vessels) in contact with the implant surface (histometric linear measurements) and within 400 μm from the implant surface (morphometric measurements) were evaluated.

For the morphometric measurements, a point counting method was applied [22], using a lattice with squares of 50 microns.

### 2.10. Data Analysis

Mean values are reported within the text, while mean values and standard deviations as well as the 25th, 50th (median), and 75th percentiles are illustrated in the tables. The primary variables were new bone for both linear and morphometric evaluations. The other variables were considered as secondary variables.

Prism 9.1.1 (GraphPad Software, LLC, San Diego, CA, USA) was used for statistical analyses. A Mann–Whitney test was used to evaluate differences between the membrane (Mb) and non-membrane (non-Mb) groups, and a Wilcoxon test was used for differences between the apical and coronal regions. The level of significance was set at α 0.05.

## 3. Results

### 3.1. Clinical Outcomes

Twenty patients were initially included in the study (Table 1). In the membrane group, one sinus membrane was perforated in a central position during the surgical procedures in the membrane group. The perforation was ~5 mm and resulted in being protected after the placement of the collagen membrane. No complications were reported by any patient.

**Figure 1:**
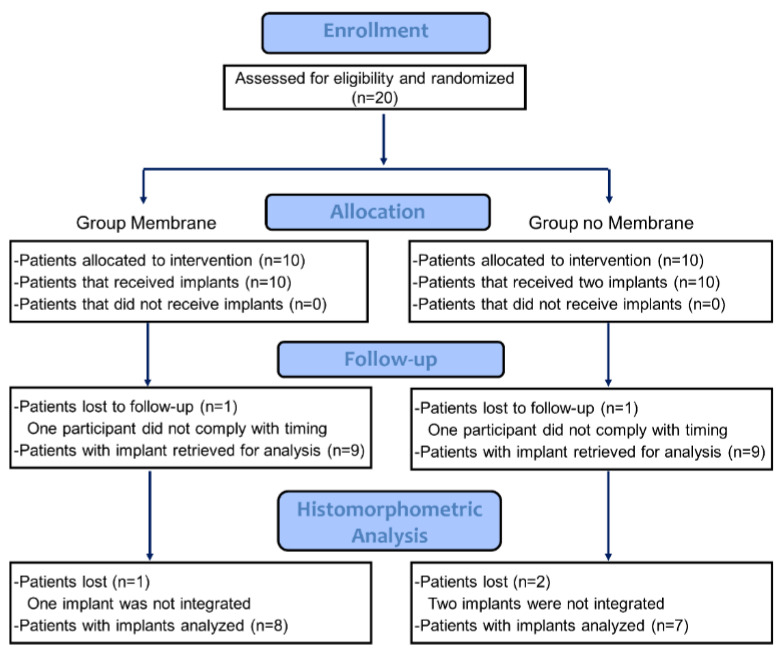
Consort flow diagram.

Two patients, one from each group, did not comply with the timing, so that both were excluded from the histological analysis (Figure 1). Three mini implants, two in the non-Mb group and one in the Mb group, were found to be not integrated. Hence, the histological analysis was performed in eight specimens from the Mb group (n = 8) and seven from the non-Mb group (n = 7).

### 3.2. Histomorphometric Description

**Figure 2:**
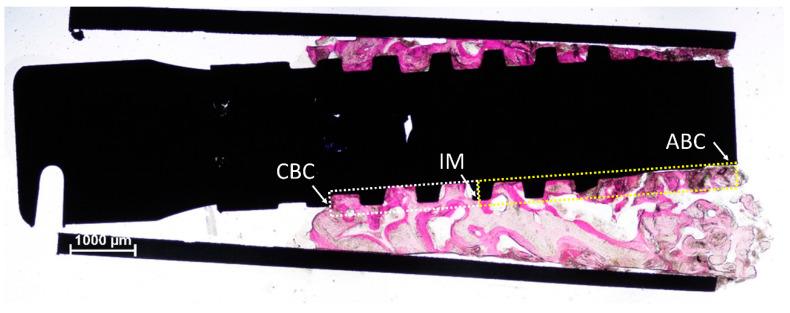
The trephine was used in an eccentric mode so that a smaller biopsy was obtained, but still providing enough tissue to be analyzed. The figure represents an example of histological measurements. The linear assessments were performed between the most coronal bone contact (CBC) to the implant surface to the half of the length of the implant (IM, intermediate point) and from IM and the most apical bone contact (ABC; white arrows). The morphometric measurements were also performed both in the coronal (white dotted lines) and apical (yellow dotted lines) regions. Acid fuchsine and toluidine blue stain.

The biopsies were retrieved with trephines used in an eccentric mode [21] that allowed a reduced dimension of the osteotomy but a collection of sufficient tissues to be analyzed (Figure 2). Mineralized bone, marrow spaces, and residues of biomaterial in contact with and around the implant surface were analyzed.

**Figure 3:**
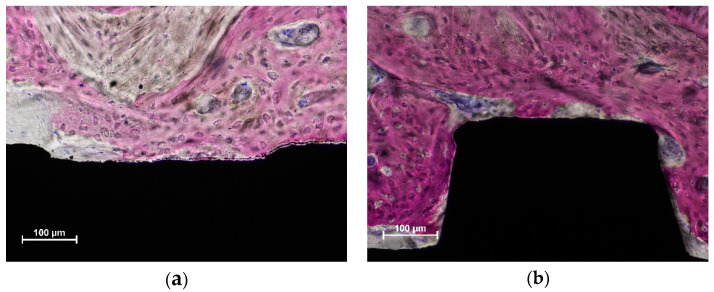
New bone was observed both in the coronal (**a**,**b**) more apical regions. Acid fuchsine and toluidine blue stain.

New bone was found in contact with and around the implant surface in both the coronal (Figure 3a) and more apical (Figure 3b) regions.

**Figure 4:**
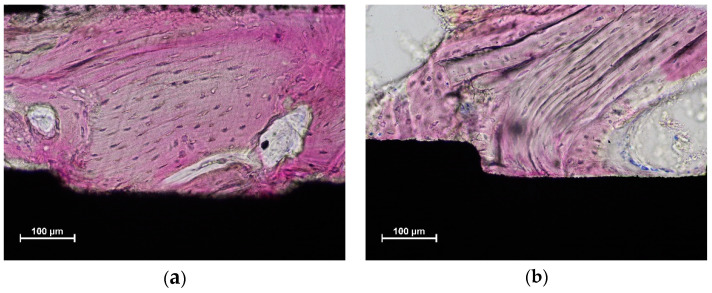
Old parent bone of the sinus floor still in contact with the implant surface in both figures (**a**,**b**). Acid fuchsine and toluidine blue stain.

In the coronal region, residues from the parent bone of the sinus floor were observed (Figure 3a), sometimes in contact with the implant surface (Figure 4a,b).

**Figure 5:**
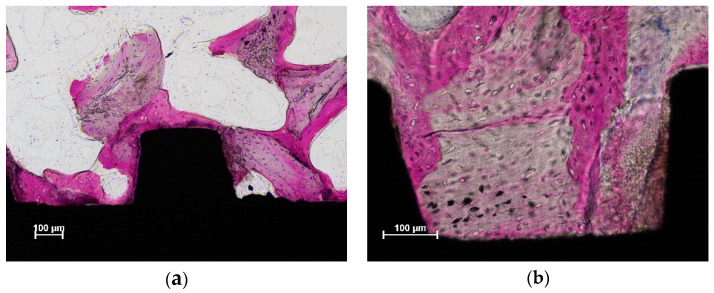
Graft particles (light purple) included into newly-formed bone (dark purple) and in contact with the implant surface in both figures (**a**,**b**). Acid fuchsine and toluidine blue stain.

Xenograft particles were included into the newly-formed bone (Figure 5a), and, in several instances, the graft was in contact with the implant surface (Figure 5a,b).

### 3.3. Histometric Evaluations—Tissues in Contact with the Implant Surface

No statistically significant differences were found between Mb and non-Mb groups for any of the tissues in contact with the implant surface (Table 2). New bone was represented by 51.3% and 52.5% at the Mb and non-Mb groups, respectively, (*p* > 0.9999). A lesser amount of residual pre-existing old bone was still observed (~4% in both groups; *p* = 0.774). Graft particles were also found in contact with the mini implant surface, reaching proportions of 3.6% in the Mb group and 4.8% in the non-Mb group (*p* = 0.881). When the apical and coronal regions were compared, again no differences were found between the Mb and non-Mb groups for the variables analyzed. However, higher new bone proportions were found in the coronal (60.2% and 58.4%) compared to the apical (44.3% and 44.8%) regions (*p* = 0.078; *p* = 0.016) for the Mb and the non- groups, respectively.

### 3.4. Morphometric Evaluations

Similar densities of new bone were found around the mini implants both in the Mb and non-Mb groups in both the coronal and apical regions (Table 3; ~31–34%).

Old bone was found in higher proportions in the cortical regions compared to the apical regions. Residual grafts particles were almost completely located in the apical regions, and higher percentages were found in the Mb group (12.7%) compared to the non-Mb group (5.5%; *p* = 0.006).

## 4. Discussion

The present study failed to show differences in bone apposition onto the implant surface and bone formation around the implant. New bone density around the implants was similar in both groups as well. The results observed suggest that the placement of a collagen membrane subjacent the sinus mucosa did not affect either the bone-to-implant contact percentage (BIC%) at the mini implants or the bone density around the mini implants.

It must be considered that the sinus mucosa possesses an innate osteogenic potential [13,14]. In an in vitro study [13], frozen sections of Schneiderian membrane from pigs were incubated with bone morphogenetic protein (BMP)-6 and BMP-7. Mineralization of the extracellular matrix, alkaline phosphatase activity, and osteocalcin expression were evaluated. The results from the study allowed the authors of [13] to conclude that the sinus mucosa contains mesenchymal cells able to provide an osteogenic differentiation in response to BMP-6 and -7 stimulation. In another study [14], the osteogenic potential was demonstrated by the alkaline phosphatase activity and mRNA expression of osteogenic markers revealed by cultured cells obtained from human sinus mucosa. Moreover, in the same study, the sinus mucosa was transplanted subcutaneously in immunodeficient mice. Ectopic new bone formation was observed after 8 weeks of healing.

Nevertheless, the conditions created by the surgical procedures might hinder that potential. In fact, after sinus floor elevation, post-surgical edema/bleeding occurs subjacent the sinus mucosa, as revealed by the increased thickness reaching 2–3 times the original dimensions [3,6,7,23,24,25]. The increased dimensions of the sinus mucosa might be already visible after 1 day [26], and they might be still present after 6 weeks of healing, as described in a clinical study that reported a reduction of ~40% in the volume compared to that observed 1 week after the sinus floor elevation [23]. It was reported that the swelling of the sinus mucosa was resolved in 96% of the sinuses [24]. Moreover, after 9 months, the sinus mucosa thickness was found to be lower than that measured before surgery [3,7,25]. Under such conditions, it might be difficult for the sinus mucosa to participate in bone formation, at least during the first weeks of healing. Indeed, in a study in monkeys [18,27], no new bone formation was observed close to the sinus mucosa during the first month of healing. Moreover, in experimental studies, the placement of a collagen membrane subjacent the sinus mucosa at the test sites did not result in differences in bone formation compared to the control sites without the collagen membrane. This was shown both in sheep after 3–4 months of healing [15,16] and in rabbits after 8 weeks of healing [17].

Another aspect that should be considered as an obstacle to bone production by the sinus mucosa is the intra-sinus pressure that will tend to allow the elevated space to return to its original position [18,28,29,30,31,32]. This phenomenon was discussed in several studies. In an experiment in rabbits [28], the ostium of a maxillary sinus was obstructed with a gelatin sponge (test site), while the other ostium was left open. The sinus mucosa was elevated anteroventrally bilaterally, and a clot was allowed to fill the elevated space. Biopsies were obtained after 1, 3, and 6 weeks of healing. After 6 weeks, the elevated space obtained at the control sinuses was almost completely lost. Conversely, in the occluded sites, the elevated space was maintained over time, and mature cortical and trabecular bone was observed. The results from that study might suggest that the use of grafts, devices, or implants aiming to maintain the elevated space over time is fundamental to allow bone formation as well as the expression of the sinus mucosa osteogenic potential. However, in an experiment in monkeys that included maxillary sinus floor elevation with simultaneous implant installation with no grafts [18], no new bone was found close to the sinus mucosa 30 days after surgery. The mucosa was found collapsed onto the implant apex, preventing bone formation in that area. It might be argued that the use of a filler might have contributed to maintaining the elevated space and allowing the sinus mucosa to express its own osteogenic potential. However, the contribution to bone formation of the sinus mucosa might be limited, if it exists. In an experiment on sinus augmentation in rabbits [33], deproteinized bovine bone mineral (DBBM) of two different dimensions, either 0.250–1.0 mm or 1.0–2.0 mm, was used to fill the elevated spaces. Three periods of healing were analyzed, i.e., 2, 4, and 8 weeks. At both 4 and 8 weeks of healing, the lower content of new bone was found in the areas subjacent the sinus mucosa. This does not exclude the contribution of the sinus mucosa in bone formation. However, the authors showed that the integration of new bone to the graft was proceeding from the bone walls toward the regions furthest from that source of new bone.

Further analyses of the same material [34] identified another problem that might compromise the expression of the potential of bone formation by the sinus mucosa. In fact, a progressive thinning and perforations of the sinus mucosa were identified in regions in contact with the sharp edges of the graft particles. Three hundred and fifty-two thinned zones (<50 µm) and 17 perforations were identified in the 36 sinuses included in the experiment. The thinned mucosa presented a progressive deterioration of the structures. In the initial phases, a decreased width of the lamina propria with displacement of blood vessels and mucous glands was observed. Subsequently, the pseudostratified ciliated columnar epithelium became thinner and disappeared in the most advanced situations. The sinus mucosa was found perforated on the sharp edges of the granules that were protruding beyond the dome profile of the elevated space. The perforations increased in number over time; there was only 1 after 2 weeks of healing, and there were 13 after 8 weeks of healing. Given that the examined histological slides in that experiment in rabbits [34] only represented a limited central region of the sinus mucosa, several further thinning sites and perforations might be expected when analyzing the other regions not included in the histological assessments. The thinning and perforation of the sinus mucosa depend on the nature of the filler used, as shown in the experiments on rabbits. A similar biomaterial but manufactured at a higher temperature compared to the DBBM discussed above, yielded similar amounts of thinning and perforations of the mucosa [35]. However, the use of bone particles as a filler considerably reduced the number of both thinning and perforated mucosa [36]. On the other hand, in that experiment, the resorption of the bone graft allowed the sinus mucosa to come into contact over time with the implant surface, producing again thinning mucosa sites and perforations in those regions. It must be considered that contact of the sinus mucosa with the implant apex and threads might result in thinning mucosa sites and perforations, as shown in a rabbit model in which implants were installed simultaneously with sinus elevation [37]. Under such conditions, it seems improbable that the sinus mucosa might provide a substantial contribution to bone formation.

Similar bone healing observed at the mini implants of both groups, and the absence of complications might provide support for the use of collagen membranes subjacent the sinus mucosa. Collagen membranes are generally used in the case of perforations occurring during surgical procedures. However, use as protection of the sinus mucosa during the first period of healing might also be taken into consideration. Indeed, a protection of the sinus mucosa over time, possibly up until the establishment of a new corticalized sinus floor, might be plausible. However, that corticalization requires several months [3,6,7,24,25] so that membranes presenting a slow resorption should be used. Further studies on this topic should be planned to evaluate their effect on bone formation as well as possible complications that can arise during healing.

The BIC% observed in the present study was similar in the Mb and non-Mb groups, respectively. Other similar studies evaluated bone healing of mini implants retrieved from sinus floor augmented sites. In those studies, it was shown that the dimensions and the positions of the access window had a scarce effect on the BIC% [19,20], while the surface characteristics yielded a strong effect on the BIC%, especially when implants were installed in composite bone, which is constituted by bone and residual graft [38].

In the present study, in the apical portion of the mini implants, higher proportions of residual graft and lower percentages of soft tissues were observed in the Mb group compared to the non-Mb group. This might be interpreted as the membrane influencing healing in these regions. The basic concepts on guided tissue regeneration were introduced in dentistry during the 1970s [39] as was the use of membranes aimed to exclude undesired cells from growing into the region to be regenerated. In the present study, however, the use of a membrane excluded a possible source of bone formation from the sinus mucosa, rich in mesenchymal cells that have the potential of forming bone [13,14]. Nevertheless, the presence of a collagen membrane subjacent the sinus mucosa did not affect the bone apposition on the implant surface and bone formation in the regions around implants, including the apical region close to the sinus mucosa. It should be considered that the mini implants were placed 6 months after sinus floor elevation so that the regenerative processes were in an advanced stage of healing, and the collagen membrane was resorbed or at least was affecting the healing less. After 3 more months, the tissue evaluation was performed on the implant surface and in a region close to the implant (400 µm). Under such conditions, bone formation was strongly influenced by the osteoconductivity of the implant surface [40], while graft and soft tissues present at the time of implant installation were mostly kept after 3 further months of healing. The results from the present study might suggest that the sinus mucosa, instead of promoting bone formation, might induce graft resorption and soft tissue formation. The reasons for such outcomes, such as mechanical or biological reasons, cannot be explained by the present report, and studies with specific aims should be performed. The present study does not supply long-term outcomes either. However, a systematic review with a meta-analysis reported that the collagen membrane is the most used method for protecting sinus mucosa perforations [41]. The same review also concluded that such perforations do not entail a risk factor for dental implant survival. This might allow one to argue that the use of collagen membranes does not affect implant survival.

Several limitations should be ascribed to the present study, such as the small sample of subjects included, the use of a single mini implant that allowed for the analysis of a limited region of the sinus, and the use of a single biomaterial, while other grafts might have yielded different results. Finally, no perforations of the sinus mucosa were intentionally performed so that the inference of the results from the present study to situations in which a perforated sinus mucosa is present should be taken with caution.

## 5. Conclusions

In conclusion, the placement of a collagen membrane subjacent the sinus mucosa did not affect bone healing at the implant surface and around the implant. The small sample limits the stregth of the conclusions.

## Figures and Tables

**Table 1:** Demographic data (n = 10).

	Gender	Age	Smokers
Mb	6 females; 4 males	56.0 ± 7.5	None
Non-Mb	7 females; 3 males	54.7 ± 11.8	None

**Table 2:** Membrane group (Mb, n = 8) and non-membrane group (non-Mb, n = 7). Tissues in contact with the implant surface expressed in percentages (%) for the whole implant surface and for the apical and coronal regions of the implant. SD, standard deviation; 25%, first percentile; 75%, third percentile; # = *p* < 0.05 between apical and coronal regions.

**Whole Surface**		**New Bone**	**Old Bone**	**Tot Bone**	**Debris**	**Graft**	**Soft Tissues**
Mb	Mean ± SD Median (25%; 75%)	51.3 ± 14.0 53.0 (44.7; 56.9)	3.8 ± 3.4 3.2 (2.3; 4.6)	55.1 ± 16.6 58.3 (45.6; 60.2)	1.8 ± 2.8 0.0 (0.0; 3.1)	3.6 ± 2.5 4.0 (1.6; 5.2)	39.5 ± 15.7 36.7 (33.6; 43.5)
Non-Mb	Mean ± SD Median (25%; 75%)	52.5 ± 16.8 50.8 (41.2; 66.0)	3.9 ± 4.8 2.2 (0.0; 6.5)	56.5 ± 16.1 53.0 (50.2; 69.7)	0.3 ± 0.9 0.0 (0.0; 0.0)	4.8 ± 5.0 4.4 (0.0; 8.5)	38.4 ± 17.2 34.8 (25.0; 48.6)
**Apical Region**		**New Bone**	**Old Bone**	**Tot Bone**	**Debris**	**Graft**	**Soft Tissues**
Mb	Mean ± SD Median (25%; 75%)	44.3 ± 21.2 36.4 (31.6; 56.1)	1.5 ± 2.8 0.0 (0.0; 1.0)	45.8 ± 23.7 36.4 (31.6; 57.1)	1.1 ± 2.2 0.0 (0.0; 0.9)	7.2 ± 5.5 7.4 (2.7; 10.9)	45.8 ± 22.0 49.1 (32.6; 58.3)
Non-Mb	Mean ± SD Median (25%; 75%)	44.9 ± 16.5 ^#^ 40.6 (37.2; 57.1)	0.0 ± 0.0 0.0 (0.0; 0.0)	44.9 ± 16.5 ^#^ 40.6 (37.2; 57.1)	0.3 ± 0.8 0.0 (0.0; 0.0)	8.7 ± 8.7 12.1 (0.0; 13.6)	46.1 ± 18.8 ^#^ 44.8 (32.2; 61.8)
**Coronal Region**		**New Bone**	**Old Bone**	**Tot Bone**	**Debris**	**Graft**	**Soft Tissues**
Mb	Mean ± SD Median (25%; 75%)	60.2 ± 10.3 60.6 (55.9; 64.1)	6.0 ± 5.2 6.2 (1.9; 8.1)	66.3 ± 12.7 63.3 (57.3; 78.0)	2.2 ± 3.8 0.0 (0.0; 3.6)	0.4 ± 1.3 0.0 (0.0; 0.0)	31.0 ± 10.9 31.4 (22.0; 39.8)
Non-Mb	Mean ± SD Median (25%; 75%)	58.4 ± 18.4 ^#^ 61.9 (43.3; 73.4)	6.0 ± 6.8 3.6 (0.0; 10.9)	64.4 ± 17.0 ^#^ 65.5 (56.2; 78.9)	0.4 ± 1.0 0.0 (0.0; 0.0)	0.0 ± 0.0 0.0 (0.0; 0.0)	35.2 ± 16.9 ^#^ 34.5 (21.1; 42.6)

**Table 3:** Membrane group (Mb, n = 8) and non-membrane group (non-Mb, n = 7). Tissue density around the implant surface expressed in percentages (%) for the whole implant surface and for the apical and coronal regions of the implant. SD, standard deviation; 25%, first percentile; 75%, third percentile; * = *p* < 0.05 between Mb and non-Mb groups; ^#^ = *p* < 0.05 between apical and coronal regions.

		**New Bone**	**Old Bone**	**Tot Bone**	**Debris**	**Graft**	**Soft Tissues**
Mb	Mean ± SD Median (25%; 75%)	33.2 ± 10.5 32.1 (25.9; 38.9)	19.8 ± 9.0 19.6 (17.0; 26.6)	53.1 ± 13.4 49.9 (44.1; 58.4)	1.8 ± 3.5 0.0 (0.0; 1.4)	12.7 ± 5.1 * 12.1 (9.5; 16.9)	32.4 ± 13.8 36.2 (24.6; 44.5)
Non-Mb	Mean ± SD Median (25%; 75%)	33.1 ± 9.6 35.4 (29.5; 39.9)	14.2 ± 13.3 10.3 (4.0; 23.4)	47.3 ± 6.2 49.4 (42.3; 52.1)	3.8 ± 6.6 0.0 (0.0; 4.8)	5.5 ± 3.0 * 5.0 (3.6; 6.2)	43.4 ± 11.9 39.3 (37.7; 54.3)
**Apical Region**		**New Bone**	**Old Bone**	**Tot Bone**	**Debris**	**Graft**	**Soft Tissues**
Mb	Mean ± SD Median (25%; 75%)	33.6 ± 9.8 28.0 (26.0; 43.4)	5.5 ± 7.6 ^#^ 0.0 (0.0; 13.3)	39.1 ± 15.0 ^#^ 33.9 (26.2; 49.1)	0.7 ± 1.4 0.0 (0.0; 0.5)	23.2 ± 6.7 * ^#^ 22.2 (19.0; 29.1)	37.0 ± 16.2 40.0 (32.6; 44.1)
Non-Mb	Mean ± SD Median (25%; 75%)	30.9 ± 11.5 34.1 (24.6; 36.7)	0.6 ± 1.7 0.0 (0.0; 0.0)	31.5 ± 12.0 ^#^ 34.1 (24.6; 38.9)	2.6 ± 6.0 0.0 (0.0; 1.0)	11.6 ± 5.6 * ^#^ 12.8 (7.9; 14.4)	54.3 ± 12.8 52.1 (45.6; 62.5)
**Coronal Region**		**New Bone**	**Old Bone**	**Tot Bone**	**Debris**	**Graft**	**Soft Tissues**
Mb	Mean ± SD Median (25%; 75%)	33.7 ± 13.3 31.3 (26.4; 39.6)	32.7 ± 13.1 ^#^ 34.4 (27.8; 42.3)	66.3 ± 14.6 ^#^ 62.8 (60.5; 72.1)	2.8 ± 6.6 0.0 (0.0; 0.9)	2.2 ± 3.3 ^#^ 0.3 (0.0; 3.4)	28.7 ± 13.7 32.1 (17.5; 37.4)
Non-Mb	Mean ± SD Median (25%; 75%)	32.9 ± 9.8 36.2 (31.0; 37.9)	24.0 ± 23.4 20.3 (5.4; 40.7)	57.0 ± 20.0 ^#^ 56.5 (46.1; 62.9)	4.1 ± 9.2 0.0 (0.0; 2.0)	0.2 ± 0.3 ^#^ 0.0 (0.0; 0.2)	38.8 ± 22.3 43.5 (22.7; 53.3)

## Data Availability

The data are available upon reasonable request.
